# The faecal bulk heterogeneity: implications for homogenisation and spot-sampling strategies for metabolomic investigations

**DOI:** 10.1007/s11306-026-02451-3

**Published:** 2026-05-24

**Authors:** Eliska Jenickova, Anna Mascellani Bergo, Chandrama Roy Chowdhury, Jaroslav Havlik

**Affiliations:** Department of Food Science, Faculty of Agrobiology, Food and Natural Resources, Czech University of Life Sciences Prague, Kamycka 129, 165 00 Prague, Czech Republic

**Keywords:** Faecal metabolomics, Sampling, Stool heterogeneity, Stool topography, Pre-analytical variability, ^1^H NMR

## Abstract

**Introduction:**

Faecal metabolomics investigates small molecules in stool to elucidate metabolic pathways and identify potential biomarkers. However, topographical heterogeneity within individual stool specimens poses a challenge for data consistency. Although this issue is well recognised, quantitative comparisons across defined stool regions, sampling depths, and whole-stool homogenates remain limited.

**Objectives:**

This pilot study aimed to (i) quantify metabolite variation across five regions (from head to tail) and different depths of the faecal bulk, and (ii) determine whether spot sampling can serve as a representative alternative to whole-stool homogenisation for faecal metabolite profiling by proton nuclear magnetic resonance (^1^H NMR) spectroscopy.

**Methods:**

Seventy-six metabolites were quantified across defined faecal regions and depths. Comparative analyses determined spatial differences in metabolite abundance and assessed the similarity between spot samples and bulk homogenates.

**Results:**

Distinct metabolic profiles were observed between the two ends of the faecal bulk. The tail region, representing more recently discharged material, exhibited higher levels of 3-(3-hydroxyphenyl)propionate (+ 57–58%) and 3-phenylpropionate (+ 50%), whereas the head region showed greater abundance of proteolysis-related metabolites such as isovalerate (+ 30%) and methionine (+ 54%). No consistent differences were detected across sampling depths. Despite these metabolite-specific differences, overall deviations from the homogenised profile were small, with mean absolute differences remaining approximately 1% or less across regions.

**Conclusion:**

Pronounced longitudinal heterogeneity exists in faecal metabolite composition along the stool axis. In this pilot cohort, tail-region spot sampling most closely represents the overall faecal metabolome and may serve as a practical, less labour-intensive alternative to whole-stool homogenisation for metabolomic studies.

**Supplementary Information:**

The online version contains supplementary material available at 10.1007/s11306-026-02451-3.

## Introduction

Metabolomics is the research field investigating small molecules produced via metabolic processes (Nicholson et al., 1999), generally metabolites with molecular masses below approximately 1,500–2,000 Da, that arise from host metabolism, diet, environmental exposures, and microbial activity (Karu et al., 2018; Kim et al., 2016). In faecal samples, these molecules include short-chain fatty acids, branched-chain fatty acids, amino acids, sugars, bile acid derivatives, indoles, lipids, and a wide range of microbially transformed compounds, making the faecal metabolome a useful functional readout of gut microbial and host metabolic processes (Karu et al., 2018). In clinical research, metabolomics has become a standard omics approach for characterising metabolic pathways, understanding dynamic changes, and uncovering potential biomarkers of diseases (Johnson et al., 2016; Patti et al., 2012). Two analytical platforms are typically used in this field — proton nuclear magnetic resonance (^1^H NMR) spectroscopy and mass spectrometry coupled with chromatographic techniques — both considered complementary with their advantages and disadvantages (González-Peña & Brennan, 2019). Specifically, ^1^H NMR spectroscopy is considered a robust method, valued for its repeatability and quantitative nature, providing a holistic view of the faecal metabolome (González-Peña & Brennan, 2019). Interest in the gut environment has increased over the past two decades due to the complex effects of its composition, functioning and dynamics (Valdes et al., 2018). The gut environment is continuously influenced by the gut microbiota and host factors such as dietary habits, genetic predisposition, and medical history, making it a valuable indicator of the host’s overall health (Christ et al., 2019; Jaimes et al., 2021; Zierer et al., 2018). Investigation of the gut environment is further crucial because of the bidirectional interaction between the host and the gut microbiota (Khine et al., 2020; Zheng et al., 2020). Faeces are frequently used for this purpose due to their complexity, non-invasive collection, and close resemblance to the dynamic gut environment (Chen et al., 2019; Karu et al., 2018).

Substantial topographical heterogeneity within faecal matter presents a well-recognised challenge, as different regions of the stool often exhibit distinct metabolomic profiles (Gratton et al., 2016; Karu et al., 2018; Liang et al., 2020; Trošt et al., 2020). These variations primarily result from progressive stages of digestion along the gastrointestinal tract (Karu et al., 2018; Tarazona Carrillo et al., 2023). Visibly, the stool is typically divided into the head (beginning part of the discharged excrement) and the tail (final part of the discharged excrement), reflecting differences in colonic transit time. The head remains in the colon longer, leading to greater water loss and more extensive microbial degradation, whereas the tail is characterised by a softer consistency and more vivid colour due to shorter residence time (Culp & Goodman, 2023; Deutsch & Stres, 2021). This topographical heterogeneity complicates efforts to generate consistent and representative data using omics approaches, including metabolomics (Gorzelak et al., 2015; Liang et al., 2020). Previous studies have demonstrated spatial heterogeneity in faecal metabolite profiles and the influence of sampling region on metabolomic readouts (Deutsch & Stres, 2021; Gratton et al., 2016; Liang et al., 2020; Trošt et al., 2020); however, a structured quantitative comparison across defined longitudinal regions, sampling depths, and whole-stool homogenates remains limited. Furthermore, it remains unclear how accurately spot sampling, which is the most commonly adopted and logistically convenient approach, captures the overall faecal metabolome (whole faecal bulk homogenisation). This gap is particularly critical in large-scale studies, where spot sampling is often preferred despite its potential limitations.

Therefore, the present pilot study aims to systematically assess metabolite variation across five defined regions (from head to tail) and at different depths within the faecal bulk. Additionally, it evaluates whether spot sampling from a defined region can provide a representative alternative to whole-stool homogenisation.

## Experimental section

### Study participants and sample collection

**Fig. 1:**
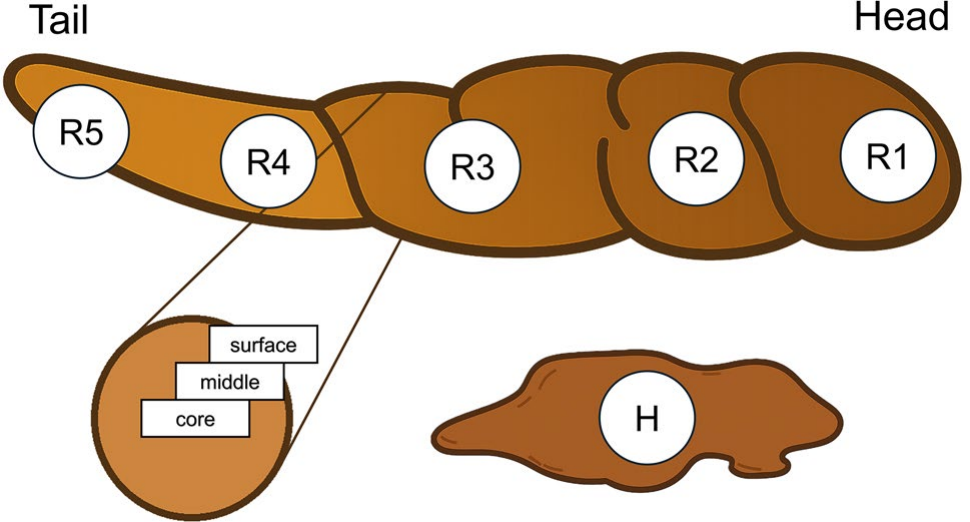
The topography of the stool samples and the description of the sampling regions. Regions (R1–R5) are defined across the faecal bulk. Head (R1) is the beginning part of the discharged excrement, tail (R5) is the final part of the discharged excrement. Surface, middle, and core subsamples were collected from the outside to the inside for each region. Homogenised matter (H) represents whole-stool homogenisation as a mixture of all regions and depths

The study was conducted with five healthy adult participants (age range between 26 and 43; 2 males and 3 females; Caucasians), all of whom were free of self-reported gastrointestinal disease, had not used antibiotics within the preceding three months, and reported no acute illness at the time of sampling. Each participant provided a complete stool specimen in an anaerobic faeces collector (Fecotainer, Gebrema, Netherlands). All samples were processed within one hour of collection, based on a previous study reporting that the faecal metabolic profile remains relatively stable within this time frame, thereby minimising potential alterations due to oxygen exposure (Gratton et al., 2016). Each faecal bulk was visually divided into five regions from which three samples were taken, each at a different depth (surface, middle, and core, Fig. 1). The rest of the bulk was mechanically homogenised in a sealed food foil (to provide mixing but prevent oxidation), and three identical samples from its centre were taken (Fig. 1). Each participant provided their faecal bulk from regular bowel movements during two separate sampling events, with an interval of at least a week between them, while following their regular diet (Table S1). Altogether, 180 samples were included in the investigation.

### Faecal metabolome analysis

The faecal aliquots were prepared according to Jaimes et al., 2021, except that 200 ± 50 mg of sample was extracted with 1 mL of ultrapure water. All chemicals and reagents used were of analytical grade and were purchased from Sigma-Aldrich (Merck, Darmstadt, Germany). The ^1^H NMR spectra were recorded on a Bruker Avance III HD spectrometer equipped with a broadband fluorine observation SmartProbe^™^ with z-axis gradients (Bruker BioSpin GmbH, Ettlingen, Germany) operating at the proton frequency of 500.18 MHz. All samples were acquired using a 1D NOESY pulse sequence with presaturation and calibrated to the internal standard (3-(trimethylsilyl) propionic-2,2,3,3-*d*_*4*_ acid sodium salt) at 0.0 ppm, manually phased in TopSpin v. 3.6.4 (Bruker Biospin GmbH). The spectra were further processed using the online software NMRProcFlow v. 1.4, in which baseline correction and manual bucketing using variable-size buckets of selected spectral regions were performed (Jacob et al., 2017). The annotated buckets in all spectra were quantified using calibration curves derived from a subset of 10 bucketed spectra and compound concentrations obtained via Chenomx software v. 8.6. Peak annotation was performed using the built-in spectral library of the Chenomx suite and our in-house database (Bervoets et al., 2021; Cui et al., 2020; Jenickova et al., 2023; Lamichhane et al., 2014). Negative values were replaced by 1/3 of the minimum positive value of each variable.

Because stool water content may differ across subjects and within longitudinal regions and sessions, normalisation to a fixed moisture content was applied. A sub-portion of faecal material from each region and of the homogenised sample (150 ± 50 mg) was lyophilised for 48 h using a laboratory freeze dryer (L4-55, Gregor Instruments, Czech Republic) to determine moisture content. Based on the measured water content of each individual sample, a sample-specific correction factor was calculated and used to normalize metabolite concentrations to a fixed water content of 70%. This value was selected as a rounded approximation of the average faecal moisture content reported in the literature (Rose et al., 2015; Schött et al., 2022; Zimmaro Bliss et al., 1999).

### Statistical and multivariate analysis

All statistical analyses were performed in Rstudio (R v. 4.3.3) (R Core Team, 2025). Exploratory and confirmatory analyses were applied to investigate metabolite variation across different faecal regions (R1–R5), homogenised faecal bulk, sampling event, and individuals.

Principal component analysis (PCA) was employed as an unsupervised method to explore global patterns, variance structure, and clustering among samples across regions, sampling event, and subjects. Data were pre-processed by mean-centring and unit variance scaling. PCA was performed using FactoMineR package v. 2.11(Lê et al., 2008) and visualised with factoextra v. 1.0.7 (Kassambara & Mundt, 2020).

Group differences in metabolite composition based on Bray–Curtis dissimilarities were tested using PERMANOVA via the *adonis2()* function in the vegan package v. 2.6–4.6 (Dinno, 2017), with 999 permutations.

To evaluate changes in metabolite composition across faecal regions while accounting for within-subject dependencies, repeated measures–ANOVA simultaneous component analysis (RM–ASCA^+^) was performed using the ALASCA package v. 1.1.3 (Jarmund et al., 2022). In the model, faecal regions were treated as fixed effects and subjects as random intercepts. Bootstrap resampling (1,000 iterations) was used for validation. The robustness of group separation and variable contributions was assessed via bootstrap-based uncertainty bars: overlapping bars for scores indicated non-significant group differences, while loadings whose confidence intervals intersected zero were considered non-significant contributors.

Metabolite-specific longitudinal changes across the faecal bulk regions were analysed using linear mixed-effects models (LMMs), with faecal region included as a fixed effect and subject as a random intercept. Each metabolite was modelled separately using the lmerTest package v. 3.1-3.1 (Kuznetsova et al., 2017). Multiple testing correction was performed using the Benjamini–Hochberg procedure, and adjusted *P* values (q-values) below 0.05 were considered statistically significant. Estimated marginal means with 95% confidence intervals (CIs) were computed and expressed as percentage changes relative to section R5 (tail), which was used as the reference level.

To evaluate the consistency of metabolite measurements across the homogenised stool sample and the five distinct regions (R1–R5), intraclass correlation coefficients (ICCs) were calculated using the irr package v. 0.84.1 (Gamer et al., 2019). For each region and donor, values were averaged across the three sampling depths; for the whole-stool homogenate, values were averaged across the three independent replicates. A two-way mixed-effects model, single-measures type, and consistency definition was applied. ICCs were interpreted as follows: < 0.40 = poor, 0.40–0.75 = fair to good, and ≥ 0.75 = excellent (Rosner, 2010). Differences in ICCs across each comparison were compared using ANOVA followed by Tukey’s HSD post hoc test. Metabolite-level deviations from the homogenised profile were further examined using linear mixed-effects models, with sample type or region as a fixed effect and subject as a random intercept. For each metabolite, model-estimated contrasts between each region and the homogenised sample were extracted, along with 95% CIs, and visualised in a forest plot. For each region, the absolute values of the model-estimated contrasts relative to the homogenised sample were averaged across all quantified metabolites to obtain the mean absolute deviation, which was used as an overall measure of agreement with the homogenised sample. For interpretability, these summary deviations were expressed as approximate relative percentage differences.

Because the study was conducted at pilot scale, simulation-based power analyses were conducted in R using the lmerTest package, with PC1 as the summary endpoint for metabolome composition. LMMs were first fitted to the observed data for (i) the comparison between homogenised and structured stool samples and (ii) the effect of sampling depth within structured stool. New datasets were then simulated across increasing subject numbers while preserving the observed within-subject sampling structure. For each sample size, the model was refitted repeatedly, and the proportion of simulations yielding a significant fixed effect at α = 0.05 was recorded as the estimated power. These estimates should be interpreted as pilot-based approximations intended to guide the design of future confirmatory studies rather than as definitive sample size recommendations.

All plots were generated using ggplot2 v. 3.5.0 (Wickham, 2016), with custom themes for clarity and consistency.

## Results

### Intra-stool spatial variation in metabolite composition

A total of 76 metabolites were annotated and quantified across all ^1^H NMR spectra, primarily comprising short-chain fatty acids (SCFAs: acetate, propionate, butyrate, valerate), branched-chain fatty acids (BCFAs: isobutyrate, isovalerate), amino acids, and mono- and disaccharides (Table S2).

**Fig. 2:**
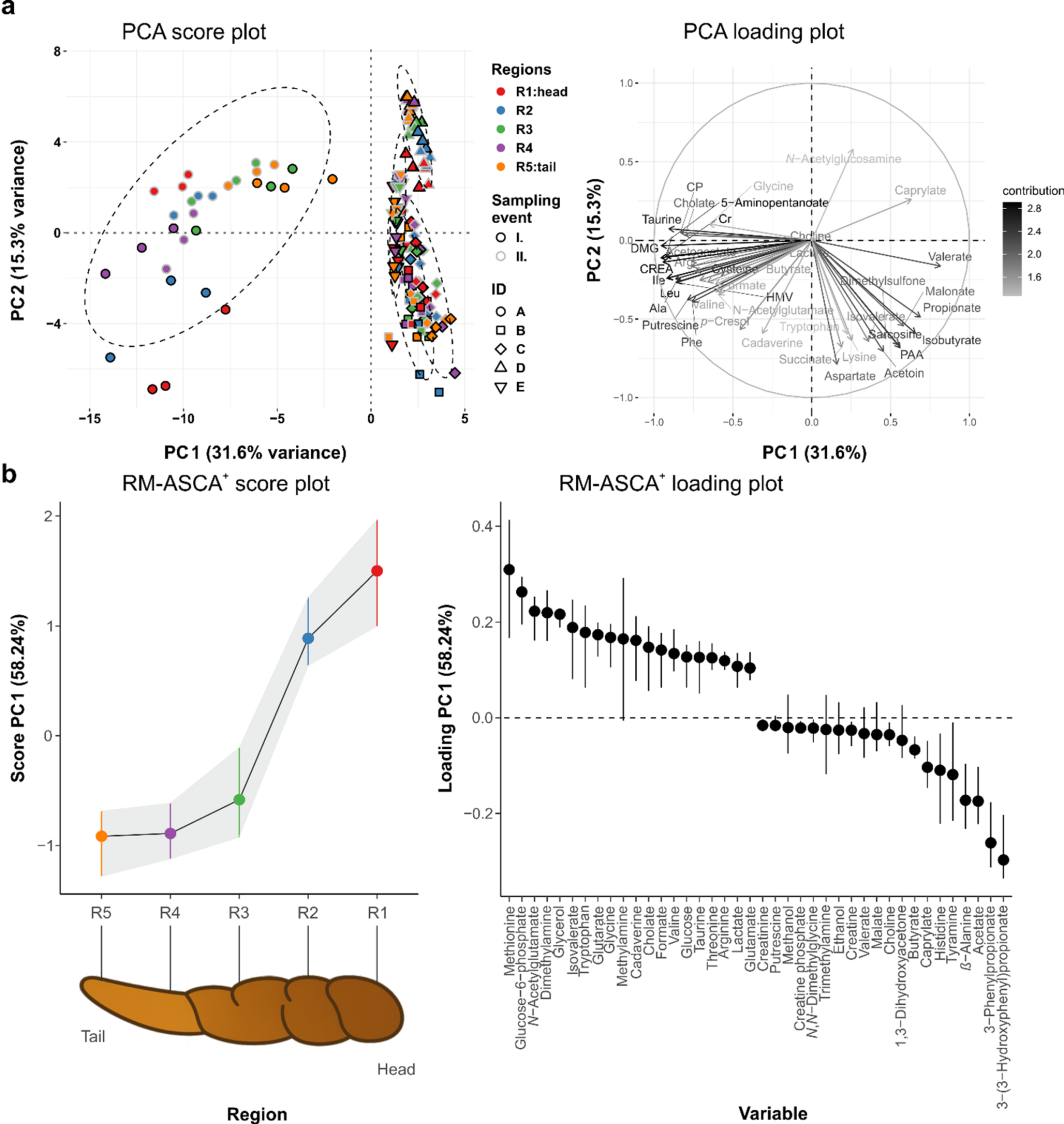
Multivariate analysis of metabolomic data. **a** Principal Component Analysis (PCA) score and loading plot illustrating the ^1^H NMR-detectable faecal metabolome variation and sample clustering in the dataset across different regions, sampling time, and individuals. Loading displays the 40 most contributing variables. **b** Repeated measures–ANOVA simultaneous component analysis (RM–ASCA^+^) score and loading plot visualising the structured variance attributable to variation across faecal topology. Loading displays the 40 most contributing variables. The head (R1) is meant as the beginning part of the discharged excrement, and the tail (R5) is the final part of the discharged excrement. *N* = 150 (regional samples; homogenised samples excluded). Ala: alanine; Arg: arginine; CP: creatine phosphate; Cr: creatinine; CREA: creatine; DMG: *N*,* N*-dimethylglycine; HMV: 2-hydroxy-3-methylvalerate; Ile: isoleucine; Leu: leucine; PAA: phenylacetate; Phe: phenylalanine

Principal component analysis (PCA) revealed considerable inter-individual variation in faecal metabolic composition (Fig. 2a). Subject A exhibited a distinct metabolomic profile, characterised by elevated levels of metabolites associated with amino acid metabolism (e.g., valine, leucine, isoleucine, phenylalanine, arginine), energy pathways (lactate, acetoacetate, creatine phosphate, creatinine), and microbiota-derived metabolites (butyrate, putrescine, cholate, taurine). High levels of choline and *N*,* N*−dimethylglycine were also detected. Subject D presented more caprylate and *N*-acetyl glucosamine than subjects B, C, and E. Together, these metabolite patterns underscored the strong subject-dependent baseline variability.

To evaluate spatial heterogeneity within the faecal bulk, we performed Bray–Curtis dissimilarity analysis and PERMANOVA, including stool region, sampling occasion, sampling depth, and subject as explanatory factors. Individual differences were the dominant source of variation, accounting for 61.2% of the total metabolomic variance (R² = 0.612, *P* = 0.001). The faecal region also contributed significantly, but with a much smaller effect size, explaining 2.2% of the variance (R² = 0.022, *P* = 0.011), indicating the presence of small but measurable intra-stool compositional gradients along the stool axis. Sampling occasion explained 2.3% of the variance (R² = 0.023, *P* = 0.001), whereas sampling depth accounted for only 0.2% and was not significant (R² = 0.002, *P* = 0.895). Thus, in contrast to the longitudinal differences observed along the stool axis, no consistent variation in NMR-detectable faecal metabolite composition was identified across the surface, middle, and core portions of the samples, indicating that sampling depth contributed minimally to intra-sample heterogeneity in this cohort.

To further characterise regional variation, we applied RM–ASCA^+^ analysis, which incorporates within-subject dependencies. This multivariate approach revealed a clear compositional gradient along the stool axis, with principal component (PC) 1 explaining 58.2% of the total variance (Fig. 2b). Metabolite concentrations changed progressively from the head to the tail. The head regions (R1–R2) were associated with relatively higher abundances of several amino acid-related and nitrogen-containing metabolites, including methionine, glucose-6-phosphate, *N*-acetylglutamate, dimethylamine, glycerol, tryptophan, glutarate, glycine, and cadaverine. In contrast, the middle (R3) and tail regions (R4 and R5), and homogenised material exhibited higher levels of microbial degradation products such as 3-(3-hydroxyphenyl)propionate, 3-phenylpropionate, acetate, *β*-alanine, tyramine, and histidine. Subsequent PCs did not reveal additional region-specific patterns.

**Fig. 3:**
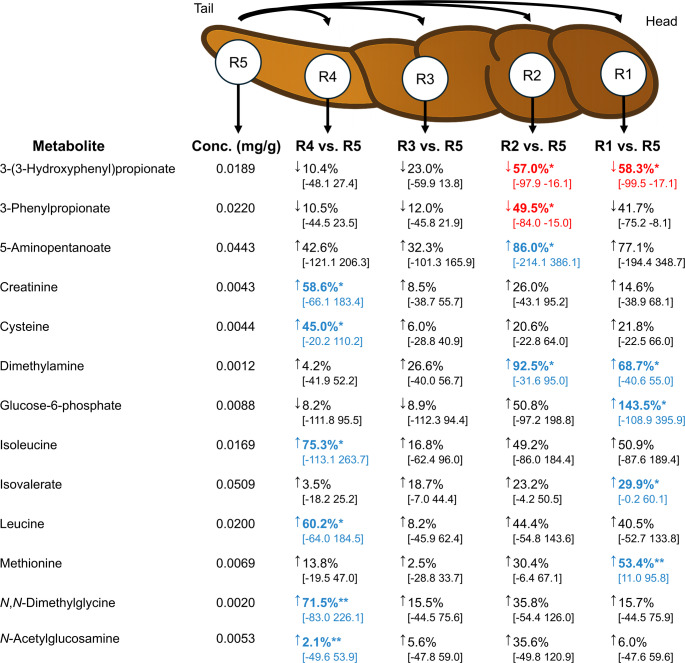
Estimated marginal mean metabolite concentrations in the faecal tail region (R5) and proportional changes observed in the other four longitudinal regions relative to R5. Statistical significance relative to the tail is indicated by asterisks (*q < 0.05, **q < 0.01, ***q < 0.001). *N* = 150 (regional samples; homogenised samples excluded). Blue upward arrows denote increases, while red downward arrows indicate decreases. Concentrations in R5 were calculated as estimated marginal means from LMMs, expressed in mg/g and standardised to a stool water content of 70%. Values inside the squared brackets represent 95% confidence interval. The head (R1) is meant as the beginning part of the discharged excrement, and the tail (R5) is the final part of the discharged excrement

While multivariate methods captured overall trends, they may underrepresent metabolites with strong localised differences and do not allow for estimating quantitative variations. To capture metabolite-specific spatial effects, we employed LMMs to directly compare concentrations between the tail and head regions of the stool. LMMs offer significant advantages over mean-based methods like t-tests or ANOVA, as they model within-subject correlation and effectively handle clustered data, which aligns with the design of the current study (Bates et al., 2015). Thirteen metabolites exhibited significant concentration differences along the longitudinal axis of the faecal bulk (Fig. 3), using region R5 (tail) as the reference. The aromatic compounds 3-(3-hydroxyphenyl)propionate and 3-phenylpropionate showed significant decreases in the head regions, with reductions of 57.0% (q = 0.017) and 58.3% (q = 0.016) in R2 and R1, respectively, for the former, and 49.5% (q = 0.025) in R2 for the latter. In contrast, 5-aminopentanoate was significantly higher in R2 (86.0%, q = 0.023). Dimethylamine concentrations increased significantly in R2 (92.5%, q = 0.002) and R1 (68.7%, q = 0.017). The BCFA isovalerate was significantly higher in R1 (29.9%, q = 0.012) and amino acid methionine was significantly increased in R1 (53.4%, q = 0.009). A strongly elevated level was also observed for glucose-6-phosphate in R1 (143.5%, q = 0.040).

Besides the differences observed between the tail (R5) and the head regions (R1 and R2), additional differences were found within the tail regions themselves, specifically between R5 and R4. In R4, significant increases were observed for creatinine (58.6%, q = 0.011), and cysteine (45.0%, q = 0.014). The branched-chain amino acids isoleucine and leucine also showed significant increases in R4 (75.3%, q = 0.039 and 60.2%, q = 0.030, respectively). Furthermore, *N*,*N*-dimethylglycine was significantly increased in R4 (71.5%, q = 0.009) as well as *N*-acetylglucosamine (2.1%, q = 0.009).

SCFAs such as acetate, butyrate, and propionate did not show significant regional differences based on LMMs. However, acetate emerged as one of the top contributors to spatial variation in the RM–ASCA^+^ model (Fig. 2b) and a marginal decrease of 9.1% in R1 (*P* = 0.044, q = 0.248), suggesting subtle but potentially relevant effects.

### Representativeness of regional sampling compared to whole-stool homogenate

**Fig. 4:**
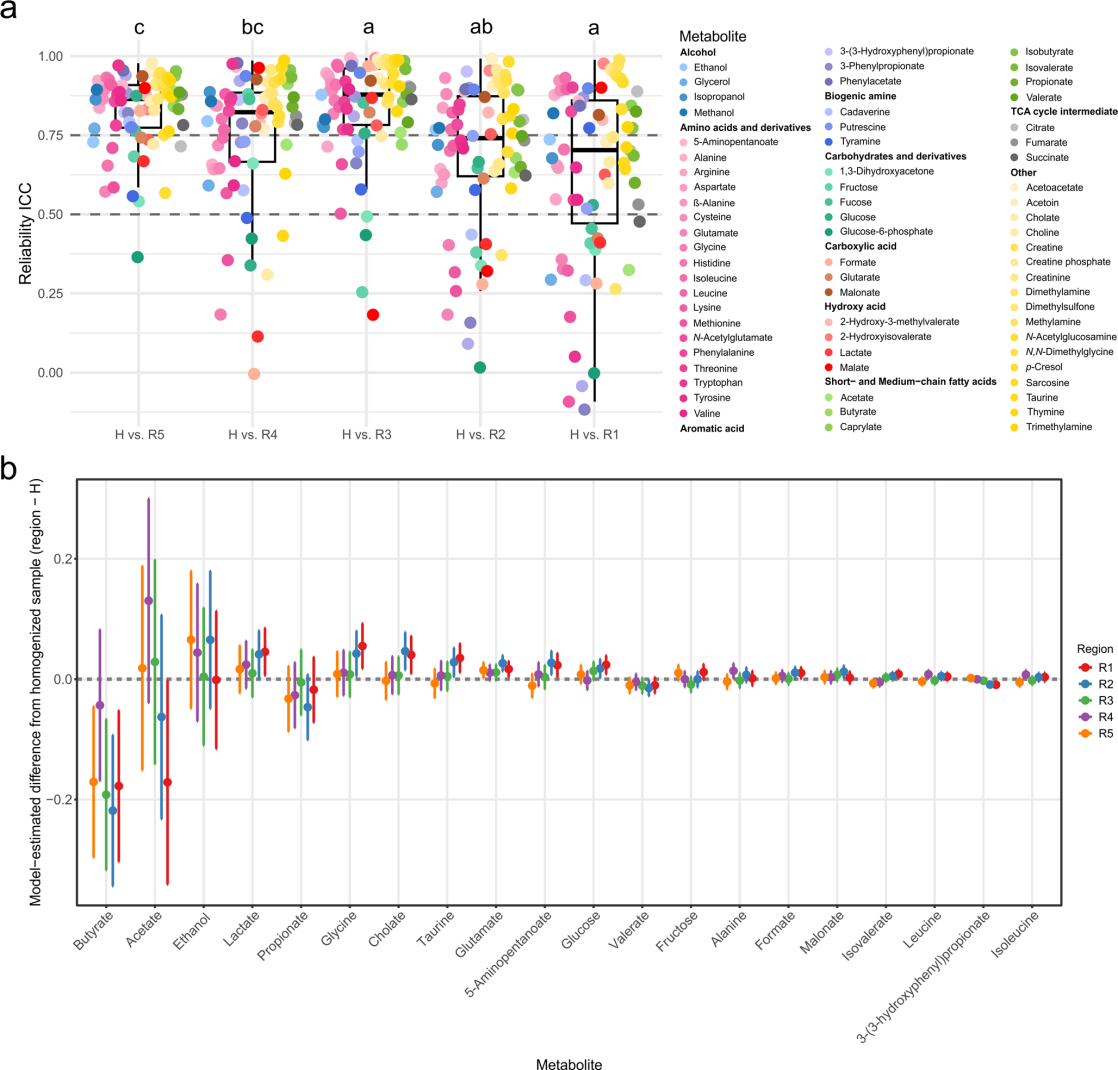
Evaluation of metabolites comparing each longitudinal region of the faecal bulk with whole-stool homogenisation. (a) Intraclass correlation coefficients (ICCs). Box plots highlight medians and IQRs. Compact letters display statistically significant output of multiple hypothesis testing using the ANOVA and Tukey’s range tests. (b) Forest plot of the top 20 metabolite-level deviations between each regional sample and the homogenised whole-stool sample. Points represent model-estimated contrasts (region − H) from linear mixed-effects models, and horizontal bars indicate 95% confidence intervals. Values closer to zero indicate closer agreement with the homogenised sample. N = 180. The head (R1) is meant as the beginning part of the discharged excrement, and the tail (R5) is the final part of the discharged excrement.

To evaluate whether regional sampling can serve as a reliable proxy for whole-stool homogenisation, the metabolite profiles of five topologically defined faecal regions were compared to those of homogenised whole-stool samples. This comparison was based on the ICC, which quantifies the degree of similarity between regional and bulk metabolite profiles. The results revealed varying levels of agreement across regions (Fig. 4a). Among all regions, the tail of the stool (R5) most closely resembled the homogenised profile, with 79.7% of the quantified metabolites showing excellent reliability (ICC > 0.75) and 18.8% good reliability (ICC 0.5–0.75). Only glucose-6-phosphate showed poor agreement in this region (ICC = 0.36). Regions R3 and R4 also demonstrated good agreement with the bulk profile (Table S3).

However, greater discrepancies emerged in regions R1 and R2 (Fig. 4a). Specifically, in R1 and R2, only 39.1% and 43.4% of the metabolites, respectively, demonstrated excellent reliability with the homogenised sample (Table S3). Despite this, medium- and short-chain fatty acids generally showed good to excellent agreement across all regions, with the exception of acetate in R1, which had poor reliability (Table S3). Most amino acids and their derivatives exhibited good to excellent agreement, although certain metabolites, including glutamate, methionine, *N*-acetylglutamate, and tryptophan showed poor agreement in R1 and R2, and lysine and glycine showed poor agreement in R1 only (Table S3). Additionally, glycine and *N*-acetylglutamate showed poor agreement in R4 (Table S3).

When evaluating the overall ICC dispersion across all metabolites, the head (R1) was the most divergent region. In contrast, R2 showed a level of similarity comparable to R4, while R3, R4, and R5 (tail) were relatively consistent with each other and similar to whole-stool homogenisation (Fig. 4a).

Because ICC reflects consistency rather than the magnitude of disagreement, we additionally examined model-estimated metabolite-level deviations between each regional sample and the homogenised stool sample (Fig. 4b). Across all quantified metabolites, the mean absolute deviation from the homogenised profile was highest for the head regions R1 (1.03%, 95% CI 0.38–1.69%) and R2 (1.02%, 95% CI 0.38–1.65%) and lower for R3 (0.49%, 95% CI 0.00–0.98%), R4 (0.55%, 95% CI 0.18–0.92%), and R5 (0.59%, 95% CI 0.12–1.07%), indicating that the central and tail regions were overall more similar to the homogenised profile than the head regions. The mean absolute deviation was approximately 1% or less in all regions. At the metabolite level, the largest deviations from the homogenised profile were observed for butyrate, acetate, lactate, glycine, propionate, ethanol, cholate, glutamate, taurine, and glucose (Fig. 4b). Butyrate showed the strongest negative deviations, particularly in R1–R3, whereas acetate was also negatively shifted, most notably in R1. In contrast, lactate, glycine, propionate, ethanol, cholate, glutamate, taurine, and glucose tended to show positive deviations, especially in R4–R5. A second group of metabolites, including 5-aminopentanoate, valerate, fructose, formate, isovalerate, malonate, alanine, and 3-(3-hydroxyphenyl)propionate, displayed smaller but still evident departures from the homogenised sample.

### Sample size estimation for future confirmatory studies

For the comparison between regional and homogenised stool samples, the estimated power was 19.0% at the current sample size of 5 subjects and increased to 80% at approximately 50 subjects. For the middle-versus-surface depth contrast, estimated power rose from 22.0% at 5 subjects to 84.5% at 30 subjects, indicating that approximately 30 subjects would be required to achieve conventional power. In contrast, the core-versus-surface comparison remained underpowered even in large simulated cohorts, consistent with the very small estimated effect size for this contrast.

## Discussion

This pilot study shows that the faecal metabolome captured by ¹H NMR varies along the longitudinal axis of the stool, whereas sampling depth contributed little to the observed variability in this cohort. Although inter-individual differences remained the dominant source of variation, clear region-specific patterns were detected within individual faecal bulks. Among the five defined regions, the tail most closely approximated the metabolite profile of the homogenised whole-stool sample, suggesting that sampling location along the stool axis is a relevant source of pre-analytical variability in faecal metabolomics.

Faecal bulk heterogeneity, characterised by substantial variation in metabolite composition, poses a significant challenge to omics-based studies (Gratton et al., 2016; Liang et al., 2020). Along the gastrointestinal tract, spatial and temporal differences in substrate availability, microbial activity, host secretions, and water reabsorption are expected to influence faecal metabolite composition (Bik et al., 2018; Procházková et al., 2023). In particular, previous literature suggests a gradual shift from predominantly saccharolytic to more proteolytic fermentation as fermentable carbohydrates become depleted distally, accompanied by changes in the production of short-chain fatty acids, branched-chain fatty acids, phenols, indoles, and polyamines (Jackson & Jewell, 2019; Korpela, 2018). In the proximal colon, saccharolysis is generally expected to predominate due to the greater availability of fermentable carbohydrates, leading to the production of SCFAs such as acetate, propionate, and butyrate. This activity is largely mediated by genera such as *Prevotella*, *Roseburia*, and *Lactobacillus* (Korpela, 2018; Vieira-Silva et al., 2016). As substrates are depleted distally, proteolysis becomes dominant, generating ammonia, hydrogen sulphide, BCFAs, phenols, indoles, and putrefactive polyamines. This metabolic shift is accompanied by changes in microbial composition, with greater abundance of *Propionibacterium*, *Clostridium*, and *Bacteroides* in the distal colon (Korpela, 2018; Pugin et al., 2017). Beyond microbial stratification, the longitudinal metabolite gradient along the stool axis is shaped by local factors such as prolonged rectal retention, increased oxygen exposure near the mucosal surface, and gradients in host secretions and water reabsorption (Albenberg et al., 2014; Chinda et al., 2022; Diether & Willing, 2019; Sheng & Hasnain, 2022). Together, these factors introduce spatial variability that complicates data interpretation, particularly in untargeted metabolomics (Gratton et al., 2016; Liang et al., 2020).

Our study quantifies region-specific metabolic variability within individual faecal samples of subjects with regular emptying in the context of ^1^H NMR metabolomics. By combining percentage changes in metabolite concentrations across defined regions, ICCs, and multivariate analyses in an untargeted ¹H NMR metabolomics approach, we provide a practical evaluation of faecal sampling strategies. The findings reveal a clear longitudinal gradient in metabolic profiles and extend previous reports of faecal spatial heterogeneity by comparing defined stool regions, sampling depths, and whole-stool homogenates within a single experimental design.

The tail—the most recently formed portion of the stool — was enriched in metabolites such as 3-(3-hydroxyphenyl)propionate and 3-phenylpropionate, known microbial catabolites of dietary polyphenols (Jiang et al., 2025; Koudoufio et al., 2020; Roager & Dragsted, 2019). This pattern is consistent with more recent digestive and microbial processing and potentially less post-excretion or post-retention transformation, although these processes were not directly assessed in the present study. In contrast, the head of the stool — representing material retained longer in the rectum — was characterised by elevated concentrations of amino acids (e.g., valine, methionine) and proteolytic catabolites such as isovalerate, isobutyrate, 5-aminopentanoate (a lysine degradation product) (Knorr et al., 2018)d acetylglutamate (from glutamine) (Shi et al., 2015). Higher glucose-6-phosphate levels in the head may also reflect greater epithelial turnover and cellular lysis (Ben-Amor et al., 2005; Van der Flier & Clevers, 2009), although this was not directly assessed in the present study. These findings are consistent with a greater relative contribution of amino acid degradation and related microbial transformations in the head region; however, this interpretation remains inferential because neither microbial composition nor functional activity were directly measured.

SCFAs concentrations remained largely consistent across regions, with only a slight trend observed for acetate. This stability reinforces the robustness of SCFAs as biomarkers of microbial activity and dietary fibre intake, supported by their consistent associations with improved metabolic health, reduced type 2 diabetes risk, and lower obesity prevalence (Cao et al., 2024; De Filippo et al., 2010; Jaimes et al., 2021; Sanna et al., 2019; Zhao et al., 2020).

Our results align with previous studies reporting spatial heterogeneity in faecal metabolite profiles (Deutsch & Stres, 2021; Gratton et al., 2016; Trošt et al., 2020). However, in comparison to reports describing depth-related gradients linked to oxygen exposure or mucosal proximity (Chinda et al., 2022; Gorzelak et al., 2015; Liang et al., 2020), we did not detect significant differences between the surface, middle, and core. Instead, heterogeneity in the present cohort was driven predominantly by longitudinal variation along the stool axis, indicating that sampling position was a more relevant source of pre-analytical variability than sampling depth. Despite regional variation, inter-individual differences remained the dominant source of variability, explaining over 60% of the total variance — consistent with previous research (Gratton et al., 2016; Liang et al., 2020). Sampling region and sampling event contributed only modestly, while sampling depth had no detectable effect. Repeated samples from the same individual clustered closely, reinforcing the short-term stability of the faecal metabolome under habitual conditions.

Although homogenisation is widely regarded as the gold standard for minimising intra-sample variability (Gorzelak et al., 2015; Gratton et al., 2016; Karu et al., 2018; Liang et al., 2020), it is rarely feasible in large-scale or home-based studies. Our findings support spot sampling from the tail as a practical and representative alternative, yielding a metabolic profile closely aligned with that of homogenised material, while avoiding the technical demands of full-sample processing. At the same time, the overall differences from the homogenised sample remained below 1% across all regions. We also observed some small but unexpected variability between adjacent tail regions (e.g., R4 vs. R5), potentially reflecting some mixing processes during defaecation or transitions from the sigmoid colon to the rectum (Palit et al., 2012). Nevertheless, the relatively consistent metabolic profile observed across regions R3 to R5 justifies using the tail as a relatively uniform and representative sampling area. This regional similarity may also explain why the homogenised sample closely resembled the tail.

While the heterogeneity of stool samples in relation to the metabolome is well known and has been investigated in previous studies (Gratton et al., 2016; Liang et al., 2020; Trošt et al., 2020), our study takes a practical approach by quantifying this phenomenon in a moderately sized experimental design. Our study provides a structured quantitative assessment of sampling-related variability in faecal metabolomics, with implications for study design, reproducibility, and biomarker interpretation. As multi-omics approaches become increasingly common in gut microbiome research, these findings have broader relevance. In DNA-based analyses, the longitudinal sample heterogeneity has a limited impact, though depth can influence the detection of mucosa-associated taxa (Gorzelak et al., 2015). RNA and protein-based methods, however, are more sensitive to sample age and location due to rapid degradation and proteolysis. Standardising the sampling region and ensuring rapid processing are therefore important for reliable transcriptomics, proteomics, and metabolomics. Whole-stool homogenisation or pooled sampling remains valuable for specific applications such as bile acid profiling, where short-term dietary effects may obscure biological signals (Matysik et al., 2016). Conversely, sampling the head may be appropriate for studies targeting rectal mucosal interactions or altered colonic motility. However, for general-purpose faecal metabolomics, our findings support spot sampling from the tail as a practical and biologically representative strategy.

However, the study is limited by the considerable variability between donors, the relatively small number of participants, and the expected variable transit time in the broader population, which was not captured in the present study. Given the small and relatively homogeneous cohort and the strong inter-individual variability observed in the dataset, the generalisability of the present findings should be considered with caution. The extent and pattern of faecal bulk heterogeneity may differ in other populations, particularly in clinical cohorts, infants, or older adults, in whom gastrointestinal transit time, stool consistency, diet, medication use, and microbiota composition may differ substantially from those of the healthy adults studied here. Such factors may influence both longitudinal and radial gradients of metabolites within the stool. Therefore, the present findings should be viewed as an initial framework that requires validation in larger and more diverse populations. The exploratory power analysis further supports interpreting the present dataset as a pilot in nature. Under the fitted model, approximately 50 subjects would be required to detect the overall difference between regional and homogenised stool samples. In contrast, depth-related effects appeared comparatively small, indicating that future confirmatory studies may be better focused on other sources of variation. Nonetheless, our cohort size is comparable to that of similar faecal heterogeneity investigations previously reported in the literature (Gratton et al., 2016; Trošt et al., 2020), and our conclusions are supported by consistent intra-individual trends and rigorous statistical modelling.

Another limitation of the present study is the analytical scope of ¹H NMR spectroscopy. Although ¹H NMR provides robust, reproducible, and quantitative profiling of abundant low-molecular-weight metabolites, it captures only part of the faecal metabolome. Important microbiome-related compound classes, including bile acids, indoles, and many lipid species, are more comprehensively covered by mass spectrometry-based methods. Therefore, the present conclusions should be interpreted primarily in relation to the NMR-detectable fraction of the faecal metabolome.

## Conclusion

Faecal bulk heterogeneity, characterised by variation in metabolite composition, represents a relevant source of pre-analytical variability in omics-based analyses. In the present cohort of healthy adults, this contribution was modest relative to the stronger effect of inter-individual variability, but a consistent longitudinal gradient was observed along the stool axis. The head of the specimen showed signs of prolonged microbial degradation, with a higher abundance of proteolysis-related metabolites. Although whole-stool homogenisation remains the most comprehensive approach, it is labour-intensive and often impractical in larger studies. Our findings suggest that, within the faecal metabolite profile captured by ¹H NMR spectroscopy in this pilot cohort, spot sampling from the tail provides a close approximation of the homogenised sample. Accordingly, sampling from the final part of the discharged excrement may represent a practical strategy for standardising faecal metabolomics workflows, provided that this region can be clearly identified. These findings should, however, be confirmed in larger and more diverse cohorts, including populations with altered transit time or stool consistency.

## Supplementary Information

Below is the link to the electronic supplementary material.

Supplementary Material 1

## Data Availability

The spectra Bruker file format, along with the description of the samples and the concentration values used for conducting the statistical analysis have been deposited in the Zenodo data repository: 10.5281/zenodo.15495279.
