# Supplementary material for: The faecal bulk heterogeneity: implications for homogenisation and spot-sampling strategies for metabolomic investigations
Source: Metabolomics. 2026 May 24;22(3):84. doi: 10.1007/s11306-026-02451-3 (PMC13199200; doi:10.1007/s11306-026-02451-3)
Supplement: Supplementary file 1 — Supplementary Material 1 [file 11306_2026_2451_MOESM1_ESM.pdf]

Supporting Information

The faecal bulk heterogeneity: homogenisation and spot-sampling strategies for metabolomic investigations

*Eliska Jenickova, Anna Mascellani Bergo, Chandrama Roy Chowdhury, and Jaroslav Havlik**

Department of Food Science, Faculty of Agrobiology, Food and Natural Resources, Czech University of Life Sciences Prague, Kamycka 129, 165 00 Prague, Czech Republic

*corresponding author: Jaroslav Havlík, havlik@af.czu.cz

Keywords: faecal metabolomics, sampling, bias, clinical trials, cohorts, ^{1}H NMR

Table of Contents

**Description**	**Page No.**
**Table S1**: Characteristics of participants together with dates of collection occasions.	2
**Table S2:** List of buckets.	3
**Table S3**. Intraclass correlation coefficients (ICCs) between the homogenised matter and each of the five regions of the faecal bulk.	5

Table S1 Characteristics of participants together with dates of collection occasions.

Subject	Sex	Age	Diet	Sampling event	Date of collection/measurement
A	female	26	no restrictions	I.	12/01/2022
				II.	26/01/2022
B	male	43	no restrictions	I.	27/01/2022
				II.	03/02/2022
C	male	34	lactose-free	I.	23/03/2022
				II.	30/03/2022
D	female	26	vegan	I.	25/03/2022
				II.	07/04/2022
E	female	31	no restrictions	I.	06/04/2022
				II.	13/04/2022

Table S2 List of buckets. Buckets covering spectrum region between δ_H 0.5–9.0 ppm (excluding the residual water region. δ_H 5.1–4.6 ppm) defined based on previously recorded features.

Bucket name	Annotation method	ppm start	ppm end
Cholate	1	0.708	0.744
2-Hydroxyisovalerate	1, 2	0.821	0.827
Valerate	1, 2	0.891	0.894
Isovalerate	1, 2	0.919	0.923
2-Hydroxy-3-methylvalerate	1, 2	0.940	0.945
Leucine	1, 2	0.962	0.966
Isoleucine	1, 2	1.004	1.009
Propionate	1, 2	1.071	1.078
Isopropanol	1, 2	1.183	1.185
Ethanol	1, 2	1.200	1.206
Fucose	1, 2	1.256	1.260
Caprylate	1	1.269	1.272
Lactate	1, 2	1.338	1.343
Acetoin	1	1.372	1.375
Cadaverine	1, 2	1.461	1.466
Alanine	1, 2	1.473	1.481
Lysine	1, 2	1.503	1.508
Butyrate	1, 2	1.579	1.590
5-Aminopentanoate	1, 2	1.657	1.665
Glutarate	1	1.806	1.811
Thymine	1, 2	1.873	1.878
Acetate	1, 2	1.920	1.929
Methionine	1, 2	2.140	2.144
N-Acetylglutamate	1	2.217	2.220
p-Cresol	1, 2	2.250	2.255
Glutamate	1, 2	2.357	2.362
Succinate	1, 2	2.376	2.381
Isobutyrate	1, 2	2.390	2.395
Citrate	1, 2	2.535	2.539
Methylamine	1, 2	2.604	2.611
Malate	1, 2	2.626	2.629
Aspartate	1, 2	2.709	2.714
Dimethylamine	1, 2	2.722	2.726
Sarcosine	1, 2	2.737	2.740
3-(3-Hydroxyphenyl)propionate	1	2.841	2.846
Trimethylamine	1, 2	2.877	2.882
N,*N*-Dimethylglycine	1, 2	2.922	2.926
Tyramine	1, 2	2.927	2.929
Creatine	1	3.037	3.043
Creatinine	1	3.047	3.052
Creatine phosphate	1	3.054	3.057
Putrescine	1	3.058	3.064

**Bucket name**	**Annotation method**	**ppm start**	**ppm end**
Cysteine	1	3.081	3.083
Malonate	1, 2	3.109	3.117
Dimethylsulfone	1	3.150	3.154
Tyrosine	1, 2	3.172	3.175
β-Alanine	1, 2	3.195	3.200
Choline	1, 2	3.201	3.208
Arginine	1	3.245	3.248
Glucose	1, 2	3.320	3.325
Tryptophan	1, 2	3.351	3.353
Methanol	1, 2	3.362	3.370
Taurine	1	3.425	3.432
Acetoacetate	1	3.445	3.448
N-Acetylglucosamine	1, 2	3.513	3.520
Glucose-6-phosphate	1, 3	3.528	3.532
Glycine	1, 2	3.564	3.571
Glycerol	1, 2	3.580	3.583
Fructose	1	3.585	3.588
Threonine	1, 2	3.603	3.606
Valine	1, 2	3.621	3.624
1,3-Dihydroxyacetone	1, 2	4.425	4.429
Fumarate	1, 2	6.521	6.527
3-Phenylpropionic acid	1	7.244	7.250
Histidine	1	7.256	7.261
Phenylacetate	1, 2	7.293	7.303
Phenylalanine	1, 2	7.333	7.348
Formate	1, 2	8.447	8.471

Table S3 Intraclass correlation coefficients (ICCs) between the homogenised matter and each of the five regions of the faecal bulk. ICCs were interpreted as follows: < 0.40 = poor, 0.40–0.75 = fair to good, and ≥ 0.75 = excellent.

Metabolite	H vs. R1	H vs. R2	H vs. R3	H vs. R4	H vs. R5
1,3-Dihydroxyacetone	0.39	0.34	0.49	0.66	0.54
2-Hydroxy-3-methylvalerate	0.80	0.82	0.97	0.79	0.88
2-Hydroxyisovalerate	0.98	0.95	0.99	0.82	0.83
3-(3-Hydroxyphenyl)propionate	-0.04	0.09	0.81	0.92	0.94
3-Phenylpropionate	-0.12	0.16	0.66	0.79	0.82
5-Aminopentanoate	0.86	0.89	0.99	0.97	0.91
Acetate	0.32	0.63	0.76	0.72	0.89
Acetoacetate	0.73	0.99	0.99	0.77	0.95
Acetoin	0.73	0.91	0.93	0.87	0.86
Alanine	0.88	0.64	0.95	0.66	0.92
Arginine	0.55	0.60	0.71	0.64	0.84
Aspartate	0.65	0.84	0.94	0.85	0.88
β-Alanine	0.65	0.63	0.89	0.64	0.86
Butyrate	0.70	0.68	0.72	0.91	0.84
Cadaverine	0.29	0.44	0.73	0.58	0.79
Caprylate	0.84	0.89	0.86	0.93	0.94
Cholate	0.60	0.64	0.98	0.92	0.98
Choline	0.66	0.64	0.75	0.31	0.72
Citrate	0.89	0.80	0.90	0.79	0.72
Creatine	0.97	0.88	0.97	0.85	0.94
Creatine phosphate	0.96	0.96	0.98	0.92	0.83
Creatinine	0.96	0.93	0.97	0.88	0.91
Cysteine	0.92	0.93	0.92	0.84	0.86
Dimethylamine	0.74	0.66	0.91	0.96	0.91
Dimethylsulfone	0.95	0.91	0.99	0.88	0.86
Ethanol	0.74	0.77	0.88	0.79	0.73
Formate	0.28	0.28	0.97	0.00	0.83
Fructose	0.41	0.38	0.25	0.88	0.68
Fucose	0.45	0.65	0.85	0.86	0.76
Fumarate	0.53	0.73	0.90	0.81	0.88
Glucose	0.53	0.67	0.76	0.34	0.87
Glucose-6-phosphate	0.00	0.02	0.43	0.42	0.36
Glutamate	0.36	0.18	0.82	0.65	0.57
Glutarate	0.42	0.61	0.82	0.78	0.74
Glycerol	0.29	0.57	0.78	0.59	0.77
Glycine	0.33	0.40	0.86	0.18	0.93
Histidine	0.70	0.70	0.91	0.80	0.65
Isobutyrate	0.77	0.89	0.98	0.96	0.95
Isoleucine	0.93	0.77	0.97	0.74	0.93
Isopropanol	0.68	0.72	0.78	0.89	0.86
Isovalerate	0.69	0.79	0.96	0.93	0.87
Lactate	0.41	0.41	0.87	0.11	0.67
Leucine	0.90	0.82	0.97	0.77	0.91

**Metabolite**	**H vs. R1**	**H vs. R2**	**H vs. R3**	**H vs. R4**	**H vs. R5**
Lysine	0.32	0.72	0.84	0.88	0.91
Malate	0.90	0.32	0.18	0.96	0.90
Malonate	0.81	0.87	0.92	0.93	0.94
Methanol	0.82	0.77	0.80	0.86	0.89
Methionine	-0.09	0.32	0.50	0.57	0.71
Methylamine	0.26	0.37	0.85	0.85	0.94
N,*N*-Dimethylglycine	0.99	0.94	0.97	0.84	0.91
N-Acetylglucosamine	0.94	0.80	0.87	0.92	0.95
N-Acetylglutamate	0.18	0.26	0.66	0.36	0.59
p-Cresol	0.93	0.92	0.91	0.79	0.75
Phenylacetate	0.84	0.90	0.97	0.98	0.95
Phenylalanine	0.90	0.83	0.86	0.86	0.78
Propionate	0.60	0.65	0.88	0.84	0.83
Putrescine	0.90	0.89	0.95	0.94	0.78
Sarcosine	0.92	0.98	0.99	0.93	0.88
Succinate	0.48	0.62	0.86	0.78	0.78
Taurine	0.66	0.81	0.98	0.96	0.93
Threonine	0.55	0.85	0.92	0.87	0.88
Thymine	0.64	0.58	0.83	0.43	0.76
Trimethylamine	0.71	0.76	0.99	0.99	0.92
Tryptophan	0.05	0.73	0.83	0.98	0.97
Tyramine	0.77	0.58	0.58	0.49	0.56
Tyrosine	0.65	0.72	0.77	0.59	0.89
Valerate	0.83	0.75	0.79	0.87	0.89
Valine	0.55	0.71	0.90	0.67	0.86
